# Supplementary material for: Cytokines and Venous Leg Ulcer Healing—A Systematic Review
Source: Int J Mol Sci. 2022 Jun 10;23(12):6526. doi: 10.3390/ijms23126526 (PMC9224200; doi:10.3390/ijms23126526)
Supplement: Supplementary file 1 [file ijms-23-06526-s001.zip › Supplementary Table S1_Database searches.pdf]

Supplementary Table S1. Database searches

EMBASE: 1974 to 2021 August 17

Search		Results
1	exp leg ulcer/	14,037
2	("Varicose ulcer*" or "leg ulcer*" or "venous ulcer*" or "VLU*" or "venous wound*" or "stasis ulcer*" or "crural ulcer*" or (lower extremit* adj ulcer*) or "ulcus cruris" or "ulcer cruris").mp.	18,711
3	1 or 2	18,711
4	exp cytokine/	1,654,214
5	exp growth factor/	1,038,312
6	exp biological marker/	353,491
7	cytokine*.mp.	705,876
8	interleukin*.mp.	763,076
9	growth factor*.mp.	739,406
10	biomarker*.mp.	490,812
11	IL-1*.mp.	296,698
12	IL-2*.mp.	99,191
13	IL-3*.mp.	21,164
14	IL-4*.mp.	63,421
15	IL-5*.mp.	20522
16	IL-6*.mp.	185,132
17	IL-7*.mp.	8694
18	IL-8*.mp.	56,490
19	IL-9*.mp.	3210
20	(IL1* or IL2* or IL3* or IL4* or IL5* or IL6* or IL7* or IL8* or IL9*).mp.	76,328
21	tumor necrosis factor*.mp.	435,027
22	TNF*.mp.	323,493
23	interferon-gamma*.mp.	63,860
24	IFN*.mp.	203,854
25	vascular endothelial growth factor*.mp.	84,396
26	VEGF*.mp.	12,4481
27	granulocyte-macrophage colony-stimulating factor*.mp.	49,154

28	GM-CSF*.mp.	28,201
29	granulocyte colony-stimulating factor*.mp.	60,917
30	G-CSF*.mp.	26,194
31	platelet derived growth factor*.mp.	53,374
32	PDGF*.mp.	37,223
33	transforming growth factor*.mp.	175,672
34	TGF*.mp.	140,108
35	insulin-like growth factor*.mp.	56,514
36	IGF*.mp.	82,465
37	epidermal growth factor*.mp.	209,018
38	EGF*.mp.	185,093
39	fibroblast growth factor*.mp.	92,231
40	FGF*.mp.	55,852
41	hepatocyte growth factor*.mp.	13,620
42	HGF*.mp.	17,006
43	macrophage colony-stimulating factor*.mp.	53,341
44	M-CSF*.mp.	7332
45	keratinocyte growth factor*.mp.	4266
46	KGF*.mp.	2810
47	nerve growth factor*.mp.	34,970
48	NGF*.mp.	22,275
49	S100A8*.mp.	3191
50	S100A9*.mp.	2719
51	calprotectin*.mp.	8,296
52	or/4-51	3,270,057
53	3 and 52	1679

PubMed (including MEDLINE)

Search	Results
"Varicose ulcer"[Mesh]	5040
"Varicose ulcer*" OR "leg ulcer*" OR "venous ulcer*" OR "VLU*" OR "venous wound*" OR "stasis ulcer*" OR "crural ulcer*" OR "ulcus cruris" OR "ulcer cruris"	15,801
("Varicose ulcer"[Mesh]) OR ("Varicose ulcer*" OR "leg ulcer*" OR "venous ulcer*" OR "VLU*" OR "venous wound*" OR "stasis ulcer*" OR "crural ulcer*" OR "ulcus cruris" OR "ulcer cruris")	15,801
"Cytokines"[Mesh] OR "Biomarkers"[Mesh] OR "Vascular Endothelial Growth Factors"[Mesh] OR "Platelet-Derived Growth Factor"[Mesh] OR "Transforming Growth Factors"[Mesh] OR "Somatomedins"[Mesh] OR "Epidermal Growth Factor"[Mesh] OR "Fibroblast Growth Factors"[Mesh] OR "EGF Family of Proteins"[Mesh] OR "Nerve Growth Factor"[Mesh] OR "Leukocyte L1 Antigen Complex"[Mesh]	1,573,710
"cytokine*" OR "interleukin*" OR "growth factor*" OR "biomarker*" OR "IL-1*" OR "IL-2*" OR "IL-3*" OR "IL-4*" OR "IL-5*" OR "IL-6*" OR "IL-7*" OR "IL-8*" OR "IL-9*" OR "IL1" OR "IL2" OR "IL3" OR "IL4" OR "IL5" OR "IL6" OR "IL7" OR "IL8" OR "IL9" OR "IL10*" OR "IL11*" OR "IL12*" OR "IL13*" OR "IL14*" OR "IL15*" OR "IL16*" "IL17*" OR "IL18*" OR "IL19*" OR "IL20*" OR "IL21*" OR "IL22*" OR "IL23*" OR "IL24*" OR "IL25*" OR "IL26*" OR "IL27*" OR "IL28*" OR "IL29*" OR "IL30*" OR "IL31*" OR "IL32*" OR "IL33*" OR "IL34*" OR "IL35*" OR "IL36*" OR "IL37*" OR "IL38*" OR "IL39*" OR "IL40*" OR "tumor necrosis factor*" OR "TNF" OR "interferon-gamma*" OR "IFN" OR "vascular endothelial growth factor*" OR "VEGF*" OR "granulocyte-macrophage colony-stimulating factor*" OR "GM-CSF*" OR "granulocyte colony-stimulating factor*" OR "G-CSF*" OR "platelet derived growth factor*" OR "PDGF*" OR "transforming growth factor*" OR "TGF" OR "insulin-like growth factor*" OR "IGF" OR "epidermal growth factor*" OR "EGF" OR "fibroblast growth factor*" OR "FGF" OR "hepatocyte growth factor*" OR "HGF" OR "macrophage colony-stimulating factor*" OR "M-CSF*" OR "keratinocyte growth factor*" OR "KGF" OR "nerve growth factor*" OR "NGF" OR "S100A8*" OR "S100A9*" OR "calprotectin*" OR "Calgranulin*"	903,929
("Cytokines"[Mesh] OR "Biomarkers"[Mesh] OR "Vascular Endothelial Growth Factors"[Mesh] OR "Platelet-Derived Growth Factor"[Mesh] OR "Transforming Growth Factors"[Mesh] OR "Somatomedins"[Mesh] OR "Epidermal Growth Factor"[Mesh] OR "Fibroblast Growth Factors"[Mesh] OR "EGF Family of Proteins"[Mesh] OR "Nerve Growth Factor"[Mesh] OR "Leukocyte L1 Antigen Complex"[Mesh]) OR ("cytokine*" OR "interleukin*" OR "growth factor*" OR "biomarker*" OR "IL-1*" OR "IL-2*" OR "IL-3*" OR "IL-4*" OR "IL-5*" OR "IL-6*" OR "IL-7*" OR "IL-8*" OR "IL-9*" OR "IL1" OR "IL2" OR "IL3" OR "IL4" OR "IL5" OR "IL6" OR "IL7" OR "IL8" OR "IL9" OR "IL10*" OR "IL11*" OR "IL12*" OR "IL13*" OR "IL14*" OR "IL15*" OR "IL16*" "IL17*" OR "IL18*" OR "IL19*" OR "IL20*" OR "IL21*" OR "IL22*" OR "IL23*" OR "IL24*" OR "IL25*" OR "IL26*" OR "IL27*" OR "IL28*"	1,874,870

OR "IL29*" OR "IL30*" OR "IL31*" OR "IL32*" OR "IL33*" OR "IL34*" OR "IL35*" OR "IL36*" OR "IL37*" OR "IL38*" OR "IL39*" OR "IL40*" OR "tumor necrosis factor*" OR "TNF" OR "interferon-gamma*" OR "IFN" OR "vascular endothelial growth factor*" OR "VEGF*" OR "granulocyte-macrophage colony-stimulating factor*" OR "GM-CSF*" OR "granulocyte colony-stimulating factor*" OR "G-CSF*" OR "platelet derived growth factor*" OR "PDGF*" OR "transforming growth factor*" OR "TGF" OR "insulin-like growth factor*" OR "IGF" OR "epidermal growth factor*" OR "EGF" OR "fibroblast growth factor*" OR "FGF" OR "hepatocyte growth factor*" OR "HGF" OR "macrophage colony-stimulating factor*" OR "M-CSF*" OR "keratinocyte growth factor*" OR "KGF" OR "nerve growth factor*" OR "NGF" OR "S100A8*" OR "S100A9*" OR "calprotectin*" OR "Calgranulin*")	
(("Varicose ulcer"[Mesh]) OR ("Varicose ulcer*" OR "leg ulcer*" OR "venous ulcer*" OR "VLU*" OR "venous wound*" OR "stasis ulcer*" OR "crural ulcer*" OR "ulcus cruris" OR "ulcer cruris")) AND (("Cytokines"[Mesh] OR "Biomarkers"[Mesh] OR "Vascular Endothelial Growth Factors"[Mesh] OR "Platelet-Derived Growth Factor"[Mesh] OR "Transforming Growth Factors"[Mesh] OR "Somatomedins"[Mesh] OR "Epidermal Growth Factor"[Mesh] OR "Fibroblast Growth Factors"[Mesh] OR "EGF Family of Proteins"[Mesh] OR "Nerve Growth Factor"[Mesh] OR "Leukocyte L1 Antigen Complex"[Mesh]) OR ("cytokine*" OR "interleukin*" OR "growth factor*" OR "biomarker*" OR "IL-1*" OR "IL-2*" OR "IL-3*" OR "IL-4*" OR "IL-5*" OR "IL-6*" OR "IL-7*" OR "IL-8*" OR "IL-9*" OR "IL1" OR "IL2" OR "IL3" OR "IL4" OR "IL5" OR "IL6" OR "IL7" OR "IL8" OR "IL9" OR "IL10*" OR "IL11*" OR "IL12*" OR "IL13*" OR "IL14*" OR "IL15*" OR "IL16*" "IL17*" OR "IL18*" OR "IL19*" OR "IL20*" OR "IL21*" OR "IL22*" OR "IL23*" OR "IL24*" OR "IL25*" OR "IL26*" OR "IL27*" OR "IL28*" OR "IL29*" OR "IL30*" OR "IL31*" OR "IL32*" OR "IL33*" OR "IL34*" OR "IL35*" OR "IL36*" OR "IL37*" OR "IL38*" OR "IL39*" OR "IL40*" OR "tumor necrosis factor*" OR "TNF" OR "interferon-gamma*" OR "IFN" OR "vascular endothelial growth factor*" OR "VEGF*" OR "granulocyte-macrophage colony-stimulating factor*" OR "GM-CSF*" OR "granulocyte colony-stimulating factor*" OR "G-CSF*" OR "platelet derived growth factor*" OR "PDGF*" OR "transforming growth factor*" OR "TGF" OR "insulin-like growth factor*" OR "IGF" OR "epidermal growth factor*" OR "EGF" OR "fibroblast growth factor*" OR "FGF" OR "hepatocyte growth factor*" OR "HGF" OR "macrophage colony-stimulating factor*" OR "M-CSF*" OR "keratinocyte growth factor*" OR "KGF" OR "nerve growth factor*" OR "NGF" OR "S100A8*" OR "S100A9*" OR "calprotectin*" OR "Calgranulin*"))	494

Web of Science

1.	ALL=("Varicose ulcer*" OR "leg ulcer*" OR "venous ulcer*" OR "VLU*" OR "venous wound*" OR "stasis ulcer*" OR "crural ulcer*" OR "ulcus cruris" OR "ulcer cruris")	11,234
2.	ALL=("cytokine*" OR "interleukin*" OR "growth factor*" OR "biomarker*")	1,596,567
3.	ALL=("IL-1*" OR "IL-2*" OR "IL-3*" OR "IL-4*" OR "IL-5*" OR "IL-6*" OR "IL-7*" OR "IL-8*" OR "IL-9*" OR "IL1" OR "IL2" OR "IL3" OR "IL4" OR "IL5" OR "IL6" OR "IL7" OR "IL8" OR "IL9" OR "IL10*" OR "IL11*" OR "IL12*" OR "IL13*" OR "IL14*" OR "IL15*" OR "IL16*" "IL17*" OR "IL18*" OR "IL19*" OR "IL20*" OR "IL21*" OR "IL22*" OR "IL23*" OR "IL24*" OR "IL25*" OR "IL26*" OR "IL27*" OR "IL28*" OR "IL29*" OR "IL30*" OR "IL31*" OR "IL32*" OR "IL33*" OR "IL34*" OR "IL35*" OR "IL36*" OR "IL37*" OR "IL38*" OR "IL39*" OR "IL40*")	1,923,496
4.	ALL=("tumor necrosis factor*" OR "TNF" OR "interferon-gamma*" OR "IFN" OR "vascular endothelial growth factor*" OR "VEGF*" OR "granulocyte-macrophage colony-stimulating factor*" OR "GM-CSF*" OR "granulocyte colony-stimulating factor*" OR "G-CSF*" OR "platelet derived growth factor*" OR "PDGF*" OR "transforming growth factor*" OR "TGF" OR "insulin-like growth factor*" OR "IGF" OR "epidermal growth factor*" OR "EGF" OR "fibroblast growth factor*" OR "FGF" OR "hepatocyte growth factor*" OR "HGF" OR "macrophage colony-stimulating factor*" OR "M-CSF*" OR "keratinocyte growth factor*" OR "KGF" OR "nerve growth factor*" OR "NGF" OR "S100A8*" OR "S100A9*" OR "calprotectin*" OR "Calgranulin*")	1,029,952
5.	((#2) OR #3) OR #4	3,502,195
6.	(#1) AND #5	1200

Cochrane (CENTRAL)

Search		Results
1.	MeSH descriptor: [Varicose Ulcer] explode all trees	658
2.	Varicose ulcer*" OR "leg ulcer*" OR "venous ulcer*" OR "VLU*" OR "venous wound*" OR "stasis ulcer*" OR "crural ulcer*" OR "ulcus cruris" OR "ulcer cruris"	1757
3.	{or #1-#2}	1757
4.	MeSH descriptor: [Cytokines] explode all trees	20,757
5.	MeSH descriptor: [Intercellular Signaling Peptides and Proteins] explode all trees	28,085
6.	MeSH descriptor: [Biomarkers] explode all trees	21,212
7.	cytokine* OR interleukin* OR growth factor* OR biomarker*	93, 412
8.	"IL-1*" OR "IL-2*" OR "IL-3*" OR "IL-4*" OR "IL-5*" OR "IL-6*" OR "IL-7*" OR "IL-8*" OR "IL-9*" OR "IL1" OR "IL2" OR "IL3" OR "IL4" OR "IL5" OR "IL6" OR "IL7" OR "IL8" OR "IL9" OR "IL10*" OR "IL11*" OR "IL12*" OR "IL13*" OR "IL14*" OR "IL15*" OR "IL16*" "IL17*" OR "IL18*" OR "IL19*" OR "IL20*" OR "IL21*" OR "IL22*" OR "IL23*" OR "IL24*" OR "IL25*" OR "IL26*" OR "IL27*" OR "IL28*" OR "IL29*" OR "IL30*" OR "IL31*" OR "IL32*" OR "IL33*" OR "IL34*" OR "IL35*" OR "IL36*" OR "IL37*" OR "IL38*" OR "IL39*" OR "IL40*"	2178
9.	tumor necrosis factor* OR TNF* OR interferon-gamma* OR IFN* OR vascular endothelial growth factor* OR VEGF* OR granulocyte-macrophage colony-stimulating factor* OR GM-CSF* OR granulocyte colony-stimulating factor* OR G-CSF* OR platelet derived growth factor* OR PDGF* OR transforming growth factor* OR "TGF" OR insulin-like growth factor* OR IGF* OR epidermal growth factor* OR EGF* OR fibroblast growth factor* OR FGF* OR hepatocyte growth factor* OR HGF* OR macrophage colony-stimulating factor* OR M-CSF* OR keratinocyte growth factor* OR KGF* OR nerve growth factor* OR NGF* OR S100A8* OR S100A9* OR calprotectin* OR Calgranulin*	65,763
10.	{or #4-#9}	136,361
11.	#3 and #10	142

ALL DATABASES COMBINED: 3515 (including duplicates)
