# Supplementary material for: Cytokines and Venous Leg Ulcer Healing—A Systematic Review
Source: Int J Mol Sci. 2022 Jun 10;23(12):6526. doi: 10.3390/ijms23126526 (PMC9224200; doi:10.3390/ijms23126526)
Supplement: Supplementary file 1 [file ijms-23-06526-s001.zip › Supplementary Table S2_Patient characteristics and methodology.pdf]

Supplementary Table S2. Patient characteristics and methodology

First author and year	Female	Age	Wound size (cm^2)	Wound age (mo)	ABPI	DM	Infection	Auto-immunity	Immuno-suppression	Baseline differences	Sample	Sampling time	Analysis	Protease inhibitor	Sterile filtered	Centrifuge prior freezing	Storage (°C)	Disease status at inclusion	Treatment before // during study
Beidler 2009 [1]	69%	57.9	21.5	NR <6	≥0.7 1pt <0.9	Yes	No systemic infection	No severe Immune-compromised state/history of vasculitis	No severe Immune-compromised state	NR	Biopsy	0h	Luminex ELISA (TGF-β1)	NR	NR	No	-80	Ulcer age ≤ 6mo, and no prior compression	NR, but no compression with high-strenght compression before inclusion//Debridement, polyurethane foam dressing, 3- or 4-layer compression weekly
Charles 2008 [2]	NR	NR	NR	NR >1	≥0.9	NR	NR	NR	NR	NR	Biopsy	0h	High throughout cDNA microarray	NA	NA	NA	NR	Chronic VLU, >4 w	NR // NR
Drinkwater 2003 [3]	NR	NR	NR	NR	>0.8	NR	NR	NR	NR	NR	WF and Biopsy	4h	ELISA. RT-PCR for mRNA on biopsy	NR	NR	Yes	-80	NR	"Various treatments before recruitment" //3- or 4-layer compression
Escandon 2012 [4]	NR	NR	38.3	NR ≥6	>0.7	NR	No topical AB	NR	NR	NR, likely paired data	Biopsy	0h	RT-PCR	NR	NR	NA	NR	Refractory VLU, ≥6 mo and failure to improve in 30 days with multi-layered compression	NR // Treatment 3 times/w for 4 w. Ultrasound, foam dressing, 4-layer elastic compression.
Filkor 2016 [5]	48%	66.7	23.2	NR	≥0.8	No	No	No	No	NR	PBMC	0h	QRT-PCR	NR	NR	NR	NR	NR	NR // NR
Fivenson 1997 [6]	NR	NR	26.5	NR	>0.65	NR	Course of antibiotics given at inclusion	NR	NR	NR	WF	1 w	ELISA	NR	Yes	Yes	-70	Chronic VLU	NR // A 2-w course of antibiotics given before baseline. Nonadherent dressing, hydrofoam pad. Compression bandage of zink-oxide-impregnated gauze.
Gohel 2008 [7]	46%	74	4.7	3	≥0.85	Yes	No	NR	NR	Older patients had sign. lowest increases in serum VEGF, 0 to 5 w.	WF and Serum	WF: 1.5h Serum: 0h	Sandwich ELISA	NR	NR	Yes	-80	Chronic VLU, wound age range 1-180 mo	Multilayer compression assumed // Nonadhesive dressings 4-layer compression applied by trained staff
Grandi 2018 [8]	NR	NR	3459 mm^3	NR	NR	NR	NR	NR	NR	NR, but paired data	Biopsy	0h	IHC	NA	NA	NA	NR	Chronic VLU	NR // ALA-PDT + SOC. Repeated weekly up to three times.
Harris 1995 [9]	NR	72.7	31.2	167.4	NR	NR	No (incl. CFU $<10^5$	NR	NR	Age, size or duration not sign. different	WF	4-6h	ELISA and bioassay	No	Yes	Yes	-70	Healing or nonhealing. All VLU ≥6 mo	NR // NA

							microbes/g tissue												
He 1997 [10]	NR	67.8	16.1	7 mo-30 years	>0.9	No	NR	No RA	NR	NR	Serum	0h	Sandwich enzyme immunoassay	NR	NR	Yes	-20	NR, duration 7 mo-30 years	NR // NA
Hodde 2020 [11]	NR	NR (>18)	NR (1-64)	NR >1	≥0.8	Yes	No cellulitis, osteo-myelitis, wound infection	No RA, vasculitis or connective tissue disorder	No	NR	WF	1 w	Luminex ELISA: TGF-β1	NR	NR	No, dressing frozen	-80	Chronic VLU, >1 mo and with granulation tissue. Run-in period did not allow for a more than 50% reduction in wound area over 2 w of SOC + compression.	SOC and compression // Weekly treatment with SIS wound matrix, debridement, gauze, foam occlusive dressing, 4-layer compression.
Krejner 2017 [12]	63%	68.6	59.6	NR ≥2-24	≥0.8 and ≤1.1	NR	No wound infection within 4 w	NR	NR	NR	Serum	0h	ELISA	NR	NR	NR	NR	Chronic VLU, ≥2-24 mo duration	NR // Hydrofiber foam composite dressing and multilayer compression. Wound management according to TIME and recommendations of multidisciplinary expert group.
Lagatolla 1995 [13]	NR	NR	NR	NR	NR	NR	NR	NR	NR	NR	Biopsy	NA	ELISA	NR	NR	NR	NR	NR	NR // NA
Ligi 2016 [14]	Inflam.: 69% Gran.: 44%	72	12.9	40	No arterial disease	Yes	Yes	No (according to criteria, but 2 RA pt incl.)	No	Sign. difference between groups regarding age, hypertension, hyperlipidaemia, infection and VAS-score. No difference as for sex, other comorbidities, duration or wound size.	WF	Until saturation of gauze	Luminex	No	NR	Yes	-80	Chronic non-healing VLU. Mean duration 40 mo. Scheduled for skin grafting.	NR // Surgical debridement and skin grafting
Ligi 2017 [15]	67%	73.7	10.7	41.6	No arterial disease	Yes	Yes	No (but 2 pt with RA included)	No	No sign. difference between groups regarding age, sex, comorbidities, ulcer duration, surface area. However, inflam. wounds had sign. higher rate of infection, VAS scores and	WF	Until saturation of gauze	Luminex	No	NR	Yes	-80	Chronic non-healing VLU, mean wound duration 41.6 mo. Scheduled for skin grafting.	NR // Inelastic multilayer compression. Skin grafting or foam sclerotherapy was performed after WF sampling.

										frequency of hyperlipidemia.									
McQuilling 2021 [16]	100%	62.6	8.6	12.6	>0.75 - <1.3	Yes	No	No active autoimmune or immune diseases	No	Does not appear to be differences in demographic data^ between healing vs nonhealing group	WF	0h	Multiplex MAP arrays	NR	NR	No, after freezing	-80	Chronic VLU, duration unclear. Inclusion criteria: >12 mo. Baseline data: mean 51.8 w and 46.25 w. Minimum ulcer duration /text): 8 w.	Run-in period of 7+/-3 days, needed to be treated with adequate compression // SOC incl. sharp debridement, hypothermically stored amniotic membrane and multilayer compression. Weekly treatment for up to 12 w.<br>^Age, history of tobacco use, DM, history of recurrent ulcers, BMI, ABPI, ulcer age and initial wound size.
Murphy 2002 [17]	38%	67	2.9	6	≥0.8	No	NR	No RA	NR	NR, but paired data	Serum	0h	ELISA	NR	NR	Yes	-80	Nonhealing VLU with no prior treatment	No previous treatment prior inclusion // 4-layered compression
Mwaura 2006 [18]	73%	60	NR	NR ≥2	>0.9	No	NR	No RA	No	NR	WF and Biopsy	<36h	ELISA (WF) IHC (Biopsy)	NR	NR	NR ("frozen immediatly")	-80	VLU ≥8 w duration	NR // 4-layer compression
Pukstad 2010 [19]	NR	NR	NR	NR	≥0.9	No	No	No	No	NR	WF	4h	Cytokine antibody glass array. TNF-α: bioassay	NR	NR	No	-80	Chronic VLU	NR // Standard moist wound care
Sadler 2012 [20]	33%	Low dose: 65 High dose: 67	Low dose: 7.3 High dose: 22.7	Low dose: 6.5 High dose: 4	≥0.7	Yes	NR	NR	No	NR (for outcome of interest)	WF	1h	ELISA	NR	NR	NR	-80	Non-healing VLU, no reduction in area over the preceding month of standard care.	SOC: nonadhesive dressing+two inelastic compression bandages and an elastic stocking*// SOC + low dosage or high dosage doxycycline.
Senet 2003 [21]	46%	FAP: 72.3 Placebo: 72.3	12.4	FAP: 50.6 Placebo: 70	>0.8	Yes	No ulcers requiring systemic AB	No RA or connective tissue disorder	No, systemic treatment with corticosteroids or cytotoxic drugs	NR (for outcome of interest)	WF	6h	ELISA	NR	NR	Yes	-80	Chronic VLU, ≥2 mo and no tendency of healing	Standard topical treatment and compression // Cleansed with saline. Intervention: FAP^ or placebo. Hydrocolloid dressing, graded compression (cotton bandages+elastic bandages).

Serra 2013 [22]	72%	Mino-cycline: 50.7 Control: 51.3	Mino-cycline: 13.3 Control: 11.7	NR ≥ 1.5	>0.9	NR	No bacterial infection within 6 w	No connective tissue disorders incl. RA	NR	NR (for outcome of interest)	WF and plasma	WF: 4 h Plasma: 0 h	ELISA	NR	Yes	Yes	-80	Chronic VLU, ≥6 w	NR // Either minocycline+basic treatment, or only basic treatment. Some had venous surgery. Basic treatment: third-class compression stocking for 3 mo and then second-class medical compression stocking for 2 mo.
Serra 2015 [23]	69%	Doxy-cycline: 50.5 Control: 51.3	Doxy-cycline: 12.9 Control: 11.7	NR ≥ 1.5	>0.9	NR	No bacterial infection within 6 w	No connective tissue disorders incl. RA	NR	NR (for outcome of interest)	WF and plasma	WF: 4 h Plasma: 0	ELISA	NR	Yes	Yes	-80	Chronic VLU, ≥6 w	NR // Either doxycycline + SOC, or only SOC. Third-class compression stocking for 3 mo and then second-class compression stocking for 2 mo. Vein surgery also offered for some.
Stacey 2019 [24]	50%	74.5	11.2	4.0	>0.5	Yes	NR	Yes	NR	NR	WF	1h	Multiplex ELISA	NR	NR	NR	-80	Chronic VLU, median duration 4.0 mo, range 0.75-104 mo	NR // SOC incl. compression and active or non-active dressings. Different types of compression allowed (most received long stretch bandages)
Tian 2003 [25]	38%	72	63.5	NR >3	NR	NR	NR	NR	NR	NR	Biopsy	0h	IHC	NA	NA	NA	-80 and formalin (unclear)	Chronic VLU failing treatment with compression. No size reduction over >3 mo or rapid increase. Scheduled for skin grafting.	Compression therapy // Bedrest for 2 w and 6 hourly dressings with saline compresses under admission in hospital
Trengove 2000 [26]	14%	72	45.8^	NR >3	≥0.6	NR	No if IV AB for cellulitis	NR	NR	NR, but paired data	WF	1h	ELISA and bioassay	No	Yes	Yes	-80	Nonhealing: no size reduction in 3 mo or increase in size. Scheduled for skin grafting.	Compression for 3 mo // Bedrest, 6 hourly saline compresses under admission in hospital. ^Whole study population
Wallace 1998 [27]	38%	78	46	NR >3	NR^	Yes	NR	NR	NR	NR, but paired data	WF	1h	ELISA and Bioassay	NR	NR	Yes	-80	Chronic VLU not responding to compression: no ulcer size reduction over >3 mo or increase in size. Scheduled for skin grafting.	Compression // Bedrest, 6 hourly saline compresses under admission in hospital. Îschemia allowed

Wiegand 2017 [28]	14%	60.5	NLFU: 13.8 SOC: 10.7	NFLU: 19.0 SOC 38.1	0.8-1.2	Yes	No cellulitis or osteo-myelitis	NR	NR	No sign. diff. in wound size or wound duration. No correlation between DM or adequate/ non-adequate compression in non-responders or responders.	WF Biopsy (TGF-β)	0h	Luminex TGF-β: IHC	NR	NR	NR	WF: -80 Biopsy: -80	Chronic VLU. At inclusion wound age >30 days of duration. Size reduction <30% in a 2-w run-in period.	2 w run-in period with SOC: 4-layer compression, nonactive moist wound dressings, and +/- debridement//SOC and randomization to either: 1. Noncontact low-frequency ultrasound (NFLU) three times/week or control.

*Assumed.

Abbreviations: AB = Antibiotics; ABPI = Ankle-brachial pressure index; ALA-PDT = Aminolevulinic acid photodynamic therapy; BMI = Body mass index; cDNA = Complementary DNA; CFU = Colony forming unit(s); DM = Diabetes mellitus; ELISA = Enzyme-linked immuno sorbent assay; FAP = Frozen allogenic plasma; IHC = Immunohistochemistry; Incl. = Includin/included; IQR = Interquartile range; Luminex = Multiplex Assay; Mo = Months; NA = Not applicable; NLFU = Noncontact low-frequency ultrasound; NR = Not reported; PBMC = Peripheral blood mononuclear cells; Pt = Patients; QRT-PCR = Quantitative reverse transcriptase polymerase chain reaction; RA = Rheumatoid arthritis; RT-PCR = Reverse transcriptase polymerase chain reaction; SOC = Standard of care; VAS = Visual analogue scale; VLU = Venous leg ulcer(s); WF = Wound fluid

References

1. Beidler, S.K.; Douillet, C.D.; Berndt, D.F.; Keagy, B.A.; Rich, P.B.; Marston, W.A. Inflammatory cytokine levels in chronic venous insufficiency ulcer tissue before and after compression therapy. *J Vasc Surg* **2009**, *49*, 1013-1020, doi:10.1016/j.jvs.2008.11.049.
2. Charles, C.A.; Tomic-Canic, M.; Vincek, V.; Nassiri, M.; Stojadinovic, O.; Eaglstein, W.H.; Kirsner, R.S. A gene signature of nonhealing venous ulcers: potential diagnostic markers. *J Am Acad Dermatol* **2008**, *59*, 758-771, doi:10.1016/j.jaad.2008.07.018.
3. Drinkwater, S.L.; Burnand, K.G.; Ding, R.; Smith, A. Increased but ineffectual angiogenic drive in nonhealing venous leg ulcers. *J Vasc Surg* **2003**, *38*, 1106-1112, doi:10.1016/s0741-5214(03)01053-x.
4. Escandon, J.; Vivas, A.C.; Perez, R.; Kirsner, R.; Davis, S. A prospective pilot study of ultrasound therapy effectiveness in refractory venous leg ulcers. *Int Wound J* **2012**, *9*, 570-578, doi:10.1111/j.1742-481X.2011.00921.x.
5. Filkor, K.; Németh, T.; Nagy, I.; Kondorosi, É.; Urbán, E.; Kemény, L.; Szolnoky, G. The expression of inflammatory cytokines, TAM tyrosine kinase receptors and their ligands is upregulated in venous leg ulcer patients: a novel insight into chronic wound immunity. *Int Wound J* **2016**, *13*, 554-562, doi:10.1111/iwj.12473.
6. Fivenson, D.P.; Faria, D.T.; Nickoloff, B.J.; Poverini, P.J.; Kunkel, S.; Burdick, M.; Strieter, R.M. Chemokine and inflammatory cytokine changes during chronic wound healing. *Wound Repair Regen* **1997**, *5*, 310-322, doi:10.1046/j.1524-475X.1997.50405.x.
7. Gohel, M.S.; Windhaber, R.A.; Tarlton, J.F.; Whyman, M.R.; Poskitt, K.R. The relationship between cytokine concentrations and wound healing in chronic venous ulceration. *J Vasc Surg* **2008**, *48*, 1272-1277, doi:10.1016/j.jvs.2008.06.042.
8. Grandi, V.; Bacci, S.; Corsi, A.; Sessa, M.; Puliti, E.; Murciano, N.; Scavone, F.; Cappugi, P.; Pimpinelli, N. ALA-PDT exerts beneficial effects on chronic venous ulcers by inducing changes in inflammatory microenvironment, especially through increased TGF-beta release: A pilot clinical and translational study. *Photodiagnosis Photodyn Ther* **2018**, *21*, 252-256, doi:10.1016/j.pdpdt.2017.12.012.
9. Harris, I.R.; Yee, K.C.; Walters, C.E.; Cunliffe, W.J.; Kearney, J.N.; Wood, E.J.; Ingham, E. Cytokine and protease levels in healing and non-healing chronic venous leg ulcers. *Exp Dermatol* **1995**, *4*, 342-349, doi:10.1111/j.1600-0625.1995.tb00058.x.
10. He, C.F.; Cherry, G.W.; Arnold, F. Postural vasoregulation and mediators of reperfusion injury in venous ulceration. *J Vasc Surg* **1997**, *25*, 647-653, doi:10.1016/s0741-5214(97)70290-8.
11. Hodde, J.P.; Hiles, M.C.; Metzger, D.W. Characterization of the local wound environment following treatment of chronic leg ulcers with SIS wound matrix. *J Tissue Viability* **2020**, *29*, 42-47, doi:10.1016/j.jtv.2019.12.003.

12. Krejner, A.; Litwiniuk, M.; Grzela, T. LL-37 but Not 25-Hydroxy-Vitamin D Serum Level Correlates with Healing of Venous Leg Ulcers. *Arch Immunol Ther Exp (Warsz)* **2017**, *65*, 455-461, doi:10.1007/s00005-016-0423-9.
13. Lagattolla, N.R.; Stacey, M.C.; Burnand, K.G.; Gaffney, P.G. Growth factors, tissue and urokinase-type plasminogen activators in venous ulcers. *Ann Cardiol Angeiol (Paris)* **1995**, *44*, 299-303.
14. Ligi, D.; Mosti, G.; Croce, L.; Raffetto, J.D.; Mannello, F. Chronic venous disease - Part I: Inflammatory biomarkers in wound healing. *Biochim Biophys Acta* **2016**, *1862*, 1964-1974, doi:10.1016/j.bbadis.2016.07.018.
15. Ligi, D.; Croce, L.; Mosti, G.; Raffetto, J.D.; Mannello, F. Chronic Venous Insufficiency: Transforming Growth Factor-β Isoforms and Soluble Endoglin Concentration in Different States of Wound Healing. *Int J Mol Sci* **2017**, *18*, doi:10.3390/ijms18102206.
16. McQuilling, J.P.; Carter, M.J.; Fulton, J.A.; Patel, K.; Doner, B.; Serena, T.E.; Mowry, K.C. A prospective clinical trial evaluating changes in the wound microenvironment in patients with chronic venous leg ulcers treated with a hypothermically stored amniotic membrane. *Int Wound J* **2021**, *19*, doi:10.1111/iwj.13606.
17. Murphy, M.A.; Joyce, W.P.; Condron, C.; Bouchier-Hayes, D. A reduction in serum cytokine levels parallels healing of venous ulcers in patients undergoing compression therapy. *Eur J Vasc Endovasc Surg* **2002**, *23*, 349-352, doi:10.1053/ejvs.2002.1597.
18. Mwaura, B.; Mahendran, B.; Hynes, N.; Defreitas, D.; Avalos, G.; Adegbola, T.; Adham, M.; Connolly, C.E.; Sultan, S. The impact of differential expression of extracellular matrix metalloproteinase inducer, matrix metalloproteinase-2, tissue inhibitor of matrix metalloproteinase-2 and PDGF-AA on the chronicity of venous leg ulcers. *Eur J Vasc Endovasc Surg* **2006**, *31*, 306-310, doi:10.1016/j.ejvs.2005.08.007.
19. Pukstad, B.S.; Ryan, L.; Flo, T.H.; Stenvik, J.; Moseley, R.; Harding, K.; Thomas, D.W.; Espevik, T. Non-healing is associated with persistent stimulation of the innate immune response in chronic venous leg ulcers. *J Dermatol Sci* **2010**, *59*, 115-122, doi:10.1016/j.jdermsci.2010.05.003.
20. Sadler, G.M.; Wallace, H.J.; Stacey, M.C. Oral doxycycline for the treatment of chronic leg ulceration. *Archives of dermatological research* **2012**, *304*, 487-493, doi:10.1007/s00403-011-1201-5.
21. Senet, P.; Bon, F.X.; Benbunan, M.; Bussel, A.; Traineau, R.; Calvo, F.; Dubertret, L.; Dosquet, C. Randomized trial and local biological effect of autologous platelets used as adjuvant therapy for chronic venous leg ulcers. *J Vasc Surg* **2003**, *38*, 1342-1348, doi:10.1016/s0741-5214(03)00908-x.
22. Serra, R.G., R.; Buffone, G.; Gallelli, L.; De Franciscis, S. The effects of minocycline on extracellular matrix in patients with chronic venous leg ulcers. *Acta Phlebologica* **2013**, *14*, 99-107.
23. Serra, R.; Gallelli, L.; Buffone, G.; Molinari, V.; Stillitano, D.M.; Palmieri, C.; de Franciscis, S. Doxycycline speeds up healing of chronic venous ulcers. *Int Wound J* **2015**, *12*, 179-184, doi:10.1111/iwj.12077.
24. Stacey, M.C.; Phillips, S.A.; Farrokhyar, F.; Swaine, J.M. Evaluation of wound fluid biomarkers to determine healing in adults with venous leg ulcers: A prospective study. *Wound Repair Regen* **2019**, *27*, 509-518, doi:10.1111/wrr.12723.
25. Tian, Y.W.; Stacey, M.C. Cytokines and growth factors in keratinocytes and sweat glands in chronic venous leg ulcers. An immunohistochemical study. *Wound Repair Regen* **2003**, *11*, 316-325, doi:10.1046/j.1524-475x.2003.11502.x.
26. Trengove, N.J.; Bielefeldt-Ohmann, H.; Stacey, M.C. Mitogenic activity and cytokine levels in non-healing and healing chronic leg ulcers. *Wound Repair Regen* **2000**, *8*, 13-25, doi:10.1046/j.1524-475x.2000.00013.x.
27. Wallace, H.J.; Stacey, M.C. Levels of tumor necrosis factor-alpha (TNF-alpha) and soluble TNF receptors in chronic venous leg ulcers--correlations to healing status. *J Invest Dermatol* **1998**, *110*, 292-296, doi:10.1046/j.1523-1747.1998.00113.x.
28. Wiegand, C.; Bittenger, K.; Galiano, R.D.; Driver, V.R.; Gibbons, G.W. Does noncontact low-frequency ultrasound therapy contribute to wound healing at the molecular level? *Wound Repair Regen* **2017**, *25*, 871-882, doi:10.1111/wrr.12595.
