# Supplementary material for: Cytokines and Venous Leg Ulcer Healing—A Systematic Review
Source: Int J Mol Sci. 2022 Jun 10;23(12):6526. doi: 10.3390/ijms23126526 (PMC9224200; doi:10.3390/ijms23126526)
Supplement: Supplementary file 1 [file ijms-23-06526-s001.zip › Supplementary Table S3_Outcomes and time-point of biomarker collection.pdf]

Supplementary Table S3. Outcomes and time-point of cytokine collection.

First author, year	Outcomes	Definition of outcome	Follow-up	Time-point of sampling	Paired data
Beidler 2009 [1]	a. Rapid vs delayed healer<br>b. Before vs after compression	a. Rapid healing: >40% wound size reduction after 4 w of compression<br>Delayed healing: <40% reduction after 4 w of compression<br>b. Before and after 4 w of compression with Profore/Profore lite	4 w	Baseline and 4 w	a. No<br>b. Yes
Charles 2008 [2]	Healing vs non-healing	Non-healing: >5 cm2 and present >6 mo<br>Healing: <5cm2 and present for <6 mo	NA	Baseline	No
Drinkwater 2003 [3]	Healing vs non-healing	Nonhealing: Ulcer healing within 1 year of treatment<br>Healing: Unhealed ulcers at 1 year of treatment	At least 1 year, or until healed	Baseline*	No
Escandon 2012 [4]	Correlation to healing	Correlation between cytokine change and healing (change in wound area)	4 w	Baseline and 4 w	Likely
Filkor 2016 [5]	Responder vs non-responder	Responder: ≥20% area reduction in 4 w<br>Non-responder: <20% area reduction in 4 w	4 w	Unclear	No
Fivenson 1997 [6]	Correlation to healing	The individual samples correlated to the percentage healing (change in surface area)	8 w	Baseline and weekly for 8 w	No, repeated measures
Gohel 2008 [7]	Correlation to<br>a. Ulcer size change<br>b. Baseline levels and ulcer size change	a. Correlation between cytokine change and ulcer size change, baseline vs 5 w later<br>b. Correlation between baseline levels and ulcer size change at the end of the study, baseline vs 5 w later<br>Median ulcer size reduced from 4.4 to 2.2 cm^2 after 5 weeks, although 17 of 80 ulcers increased in size.	5 w	Baseline and 5 w	Yes
Grandi 2018 [8]	Correlation to wound mean volume reduction	Unclear	Unclear, around 3 w	Baseline and repeated after 1h after irradiation	Yes
Harris 1995 [9]	Healing vs non-healing	Non-healing: NR<br>Healing: Ulcer epithelializing and granulating	NA	Baseline	No
He 1997 [10]	Correlation to healing	NR	3h 20 min	Baseline after 1 h of leg dependency	Yes
Hodde 2020 [11]	Healed vs non-healed	Healed vs non-healed at 12 w<br>Healed ulcer not defined	Up to 12 w	Weekly up to 12 w	Assumed
Krejner 2017 [12]	Poor, moderate, good and fast healing (per week)	Percent reduction from initial wound area, mean wound healing/w. Wound area performed within at least 1 mo preceding the assessment. Poor: <5% per week, Moderate: 5-10%, Good: 11-15%, Fast: >15%.	NA	Baseline*	No
Lagatolla 1995 [13]	a. Healed vs non-healed<br>b. Correlation to time to healing	Healed: Complete healing within 6 mo<br>Nonhealed: No healing within 6 mo	6 mo	Unclear	No
Ligi 2016 [14]	Inflammatory vs granulating	Clinical evaluation	NR but during admission	Baseline, repeated in some after surgical debridement	No

Ligi 2017 [15]	Inflammatory vs granulating	Clinical evaluation by medical doctors with expertise	NA	Baseline	No
McQuilling 2021 [16]	a. Healing vs non-healing<br>b. Correlation between cytokine level and percentage area change	a. Non-healing: Wounds that did not achieve >85% closure by 12 w<br>Healing: Wounds that achieved >85% closure visit 12 or were healed<br>b. Cytokine levels correlated to percent wound size reduction (wounds healing between -50 to 50%).	12 w	1, 2, 3, 4, 12 w	No, repeated measurements
Murphy 2002 [17]	Healing vs non-healing (=before & after compression)	Non-healing: NR. Compression not received.<br>Healing: Reduction in the ulcer size 4-6 w from baseline on compression. Median size reduced from 2.9 to 1.4 cm^2.	12 w	Baseline and 4-6 w	Yes
Mwaura 2006 [18]	Healing vs non-healing	Non-healing: Not fulfilling the criteria of "healing".<br>Healing: >20% decrease in ulcer area after 8 w of compression, decrease in slough and increase of healthy granulation tissue.	8 w	Baseline	No
Pukstad 2010 [19]	Healing vs non-healing	NR	8 w, or healing	Baseline, 4 and 8 w	No, repeated measurements
Sadler 2012 [20]	Percentage reduction in ulcer area	Percentage reduction in ulcer area over 4 w (linear regression)	4 w	Baseline and 4 w (4 w for analysis)	No
Senet 2003 [21]	Healing vs non-healing	NR (linear healing of the wound edge calculated)	12 w	Baseline, 4, 8, 12 w	Uncertain
Serra 2013 [22]	High-healing vs slow-healing	High-healing: ≥1 cm^2/week<br>Slow-healing: <1 cm^2/week<br>Calculated by subtracting the final ulcer area at the end of the study from the initial area, and divided by no of observations.	Median follow-up time 13 mo	Baseline, w 4, 3 mo and 5 mo	Unclear, repeated measurements
Serra 2015 [23]	High-healing vs slow-healing	High-healing: ≥1 cm^2/week<br>Slow-healing: <1 cm^2/week<br>See Serra 2013 for definition of healing	Median follow-up time 13 mo	Baseline, 4 w, 3 mo, 5 mo	Unclear, repeated measurements
Stacey 2019 [24]	Healing vs non-healing (and indeterminate)	Area change over 3 w determined if the wound was healing or not healing. Non-healing: Area increase for two consecutive weeks (two measurements). The area at the middle time point of the 3 w was classified as non-healing.<br>Healing: Area reduction over two weekly segments.<br>Indeterminate: Wound showing no consistency in area reduction/increase.	Up to 13 w	Baseline and weekly up to 13 w	No, repeated measurements
Tian 2003 [25]	Healing vs non-healing	Non-healing: Chronic VLU failing outpatient treatment with compression. Pt admitted for split skin grafting. No reduction in size over >3 mo or rapid increase in size.<br>Healing: After 2 w of bed resting, formation of granulation tissue, epithelialization and reduction in size. Median size reduction: 20%.	2 w	Baseline and 2 w	Yes
Trengove 2000 [26]	a. Healing vs non-healing<br>b. Ulcer size change	a. Non-healing: No ulcer size reduction in 3 mo on compression, or increase in size at inclusion. Pt admitted for split skin grafting. Healing: Increased granulation, epithelization, reduction of slough and ulcer area after 2 w. Median size reduction: 12%.<br>b. Percentage change in wound size after 2 w.	2 w	Baseline and 2 w	Yes
Wallace 1998 [27]	Healing vs non-healing	Non-healing: No ulcer size reduction >3 mo on compression, or increase in size at inclusion.	2 w	Baseline and 2 w	Yes

		Healing: NR (2 w of regular saline dressings and bed rest: median size reduction: 8%).			
Wiegand 2017 [28]	a. Correlation to wound size reduction<br>b. Responder vs non-responder	a. Cytokine changes from baseline to w 2 correlated to percentage reduction of wound size at w 4 (prediction of healing)<br>b. Responder: >40% area reduction w 4<br>Non-responder: ≤40% area reduction w 4	4 w	Baseline, 2, 4 w	No

*Assumed

References

1. Beidler, S.K.; Douillet, C.D.; Berndt, D.F.; Keagy, B.A.; Rich, P.B.; Marston, W.A. Inflammatory cytokine levels in chronic venous insufficiency ulcer tissue before and after compression therapy. *J Vasc Surg* **2009**, *49*, 1013-1020, doi:10.1016/j.jvs.2008.11.049.
2. Charles, C.A.; Tomic-Canic, M.; Vincek, V.; Nassiri, M.; Stojadinovic, O.; Eaglstein, W.H.; Kirsner, R.S. A gene signature of nonhealing venous ulcers: potential diagnostic markers. *J Am Acad Dermatol* **2008**, *59*, 758-771, doi:10.1016/j.jaad.2008.07.018.
3. Drinkwater, S.L.; Burnand, K.G.; Ding, R.; Smith, A. Increased but ineffectual angiogenic drive in nonhealing venous leg ulcers. *J Vasc Surg* **2003**, *38*, 1106-1112, doi:10.1016/s0741-5214(03)01053-x.
4. Escandon, J.; Vivas, A.C.; Perez, R.; Kirsner, R.; Davis, S. A prospective pilot study of ultrasound therapy effectiveness in refractory venous leg ulcers. *Int Wound J* **2012**, *9*, 570-578, doi:10.1111/j.1742-481X.2011.00921.x.
5. Filkor, K.; Németh, T.; Nagy, I.; Kondorosi, É.; Urbán, E.; Kemény, L.; Szolnoky, G. The expression of inflammatory cytokines, TAM tyrosine kinase receptors and their ligands is upregulated in venous leg ulcer patients: a novel insight into chronic wound immunity. *Int Wound J* **2016**, *13*, 554-562, doi:10.1111/iwj.12473.
6. Fivenson, D.P.; Faria, D.T.; Nickoloff, B.J.; Poverini, P.J.; Kunkel, S.; Burdick, M.; Strieter, R.M. Chemokine and inflammatory cytokine changes during chronic wound healing. *Wound Repair Regen* **1997**, *5*, 310-322, doi:10.1046/j.1524-475X.1997.50405.x.
7. Gohel, M.S.; Windhaber, R.A.; Tarlton, J.F.; Whyman, M.R.; Poskitt, K.R. The relationship between cytokine concentrations and wound healing in chronic venous ulceration. *J Vasc Surg* **2008**, *48*, 1272-1277, doi:10.1016/j.jvs.2008.06.042.
8. Grandi, V.; Bacci, S.; Corsi, A.; Sessa, M.; Puliti, E.; Murciano, N.; Scavone, F.; Cappugi, P.; Pimpinelli, N. ALA-PDT exerts beneficial effects on chronic venous ulcers by inducing changes in inflammatory microenvironment, especially through increased TGF-beta release: A pilot clinical and translational study. *Photodiagnosis Photodyn Ther* **2018**, *21*, 252-256, doi:10.1016/j.pdpdt.2017.12.012.
9. Harris, I.R.; Yee, K.C.; Walters, C.E.; Cunliffe, W.J.; Kearney, J.N.; Wood, E.J.; Ingham, E. Cytokine and protease levels in healing and non-healing chronic venous leg ulcers. *Exp Dermatol* **1995**, *4*, 342-349, doi:10.1111/j.1600-0625.1995.tb00058.x.
10. He, C.F.; Cherry, G.W.; Arnold, F. Postural vasoregulation and mediators of reperfusion injury in venous ulceration. *J Vasc Surg* **1997**, *25*, 647-653, doi:10.1016/s0741-5214(97)70290-8.
11. Hodde, J.P.; Hiles, M.C.; Metzger, D.W. Characterization of the local wound environment following treatment of chronic leg ulcers with SIS wound matrix. *J Tissue Viability* **2020**, *29*, 42-47, doi:10.1016/j.jtv.2019.12.003.
12. Krejner, A.; Litwiniuk, M.; Grzela, T. LL-37 but Not 25-Hydroxy-Vitamin D Serum Level Correlates with Healing of Venous Leg Ulcers. *Arch Immunol Ther Exp (Warsz)* **2017**, *65*, 455-461, doi:10.1007/s00005-016-0423-9.
13. Lagattolla, N.R.; Stacey, M.C.; Burnand, K.G.; Gaffney, P.G. Growth factors, tissue and urokinase-type plasminogen activators in venous ulcers. *Ann Cardiol Angeiol (Paris)* **1995**, *44*, 299-303.
14. Ligi, D.; Mosti, G.; Croce, L.; Raffetto, J.D.; Mannello, F. Chronic venous disease - Part I: Inflammatory biomarkers in wound healing. *Biochim Biophys Acta* **2016**, *1862*, 1964-1974, doi:10.1016/j.bbadis.2016.07.018.
15. Ligi, D.; Croce, L.; Mosti, G.; Raffetto, J.D.; Mannello, F. Chronic Venous Insufficiency: Transforming Growth Factor-β Isoforms and Soluble Endoglin Concentration in Different States of Wound Healing. *Int J Mol Sci* **2017**, *18*, doi:10.3390/ijms18102206.

16. McQuilling, J.P.; Carter, M.J.; Fulton, J.A.; Patel, K.; Doner, B.; Serena, T.E.; Mowry, K.C. A prospective clinical trial evaluating changes in the wound microenvironment in patients with chronic venous leg ulcers treated with a hypothermically stored amniotic membrane. *Int Wound J* **2021**, *19*, doi:10.1111/iwj.13606.
17. Murphy, M.A.; Joyce, W.P.; Condron, C.; Bouchier-Hayes, D. A reduction in serum cytokine levels parallels healing of venous ulcers in patients undergoing compression therapy. *Eur J Vasc Endovasc Surg* **2002**, *23*, 349-352, doi:10.1053/ejvs.2002.1597.
18. Mwaura, B.; Mahendran, B.; Hynes, N.; Defreitas, D.; Avalos, G.; Adegbola, T.; Adham, M.; Connolly, C.E.; Sultan, S. The impact of differential expression of extracellular matrix metalloproteinase inducer, matrix metalloproteinase-2, tissue inhibitor of matrix metalloproteinase-2 and PDGF-AA on the chronicity of venous leg ulcers. *Eur J Vasc Endovasc Surg* **2006**, *31*, 306-310, doi:10.1016/j.ejvs.2005.08.007.
19. Pukstad, B.S.; Ryan, L.; Flo, T.H.; Stenvik, J.; Moseley, R.; Harding, K.; Thomas, D.W.; Espevik, T. Non-healing is associated with persistent stimulation of the innate immune response in chronic venous leg ulcers. *J Dermatol Sci* **2010**, *59*, 115-122, doi:10.1016/j.jdermsci.2010.05.003.
20. Sadler, G.M.; Wallace, H.J.; Stacey, M.C. Oral doxycycline for the treatment of chronic leg ulceration. *Archives of dermatological research* **2012**, *304*, 487-493, doi:10.1007/s00403-011-1201-5.
21. Senet, P.; Bon, F.X.; Benbunan, M.; Bussel, A.; Traineau, R.; Calvo, F.; Dubertret, L.; Dosquet, C. Randomized trial and local biological effect of autologous platelets used as adjuvant therapy for chronic venous leg ulcers. *J Vasc Surg* **2003**, *38*, 1342-1348, doi:10.1016/s0741-5214(03)00908-x.
22. Serra, R.G., R.; Buffone, G.; Gallelli, L.; De Franciscis, S. The effects of minocycline on extracellular matrix in patients with chronic venous leg ulcers. *Acta Phlebologica* **2013**, *14*, 99-107.
23. Serra, R.; Gallelli, L.; Buffone, G.; Molinari, V.; Stillitano, D.M.; Palmieri, C.; de Franciscis, S. Doxycycline speeds up healing of chronic venous ulcers. *Int Wound J* **2015**, *12*, 179-184, doi:10.1111/iwj.12077.
24. Stacey, M.C.; Phillips, S.A.; Farrokhyar, F.; Swaine, J.M. Evaluation of wound fluid biomarkers to determine healing in adults with venous leg ulcers: A prospective study. *Wound Repair Regen* **2019**, *27*, 509-518, doi:10.1111/wrr.12723.
25. Tian, Y.W.; Stacey, M.C. Cytokines and growth factors in keratinocytes and sweat glands in chronic venous leg ulcers. An immunohistochemical study. *Wound Repair Regen* **2003**, *11*, 316-325, doi:10.1046/j.1524-475x.2003.11502.x.
26. Trengove, N.J.; Bielefeldt-Ohmann, H.; Stacey, M.C. Mitogenic activity and cytokine levels in non-healing and healing chronic leg ulcers. *Wound Repair Regen* **2000**, *8*, 13-25, doi:10.1046/j.1524-475x.2000.00013.x.
27. Wallace, H.J.; Stacey, M.C. Levels of tumor necrosis factor-alpha (TNF-alpha) and soluble TNF receptors in chronic venous leg ulcers--correlations to healing status. *J Invest Dermatol* **1998**, *110*, 292-296, doi:10.1046/j.1523-1747.1998.00113.x.
28. Wiegand, C.; Bittenger, K.; Galiano, R.D.; Driver, V.R.; Gibbons, G.W. Does noncontact low-frequency ultrasound therapy contribute to wound healing at the molecular level? *Wound Repair Regen* **2017**, *25*, 871-882, doi:10.1111/wrr.12595.
