# Supplementary material for: Cytokines and Venous Leg Ulcer Healing—A Systematic Review
Source: Int J Mol Sci. 2022 Jun 10;23(12):6526. doi: 10.3390/ijms23126526 (PMC9224200; doi:10.3390/ijms23126526)
Supplement: Supplementary file 1 [file ijms-23-06526-s001.zip › Supplementary Table S4_Overview of results for each study.pdf]

Supplementary Table S4. Overview of results for each study.

First author year, country	Study design	Outcomes	Follow-up	Specimen	Analysis	Cytokines	Main findings
Beidler 2009 [1], USA	Cohort study	a. Rapid vs. delayed healing *(>40% vs. <40% area reduction)* b. Before vs. after compression	4 w	Biopsy	Luminex & ELISA (TGF-β1)	G-CSF, GM-CSF, IFN-γ, IL-1α, IL-1β, IL1Ra, IL-2, IL-4, IL-5, IL-6, IL-7, IL-8, IL-10, IL12p40, IL-12p70, IL-13, IL-15, IL-17, TGF-β1, TNF-α	a. ↑ GM-CSF, IFN-γ, IL-1α, IL-1β, IL12p40, *before* compression in rapid healing vs. delayed. ↑ IL-1Ra *after* compression in rapid healing. b. ↑ G-CSF, GM-CSF, IFN-γ, IL-1α, IL-1β, IL-6, IL-8, IL12p40, TNF-α before compression vs. after. ↑ TGF-β1 after compression and trend for ↑IL-10 (p=0.076).
Charles 2008 [2], USA	Cross-sectional study	Healing vs. non-healing	NA	Biopsy	High throughout cDNA microarray	HB-EGF, PDGFRα, S100A7	↓ S100A7 at the edge of non-healing VLU, and HB-EGF in the ulcer bed. ↑ PDGF receptor in the bed of non-healing.
Drinkwater 2003 [3], UK	Cohort study	Healing vs. non-healing	At least 1 year, or until healed	WF, biopsy	ELISA, RT-PCR	ELISA: VEGF-165 (WF, biopsy), VEGF-R1 (WF). mRNA biopsy: VEGF-121, VEGF-165, VEGF-189, VEGF-R1, VEGF-R2	↑ VEGF-165 in WF in non-healing vs. healing, but not in biopsy. Non-healing wounds have a high level of expression of VEGF, at both the gene transcript and protein level. Results for mRNA cannot be reliably extracted
Escandon 2012 [4], USA	Clinical trial: Ultrasound	Correlation between change in cytokine level and healing (wound area change)	4 w	Biopsy	RT-PCR	IL-1α, IL-6, IL-8, IL-10, IL-11, TNF-α, VEGF	↓ IL-1α and TNF-α with healing (p<0.05, and a trend for reduction of IL-6 (p=0.0508), IL-8 (p=0.099), IL-11 (p=0.082) and VEGF (p=0.066).
Filkor 2016 [5], Hungary	Cohort study (unclear if prospective)	Responding vs. non-responding (≥*20% vs <20% area reduction)*	4 w	PBMC	QRT-PCR	IL-1α, IL-8, IL-10, TNF-α,	↑ IL-1α and IL-8 in non-responders
Fivenson 1997 [6], USA	Cohort study	Correlation to healing	8 w	WF	ELISA	IL-8, IL-10 (other cytokines cannot be reliably extracted)	↑ IL-8 with healing w 8 (p=0.09). A dynamic cytokine profile through the healing process. IL-10 increased after w 3 (11 .2% median healing), but was NS compared to baseline.
Gohel 2008 [7], UK	Cohort study	Correlation a. Between cytokine change and ulcer size change b. To baseline levels and ulcer size change	5 w	WF Serum	Sandwich ELISA	FGF-2, IL-1β, TGF-β,1 TNF-α, VEGF	a. ↑ TGF-β1 correlated to healing b. ↔
Grandi 2018 [8], Italy	Clinical trial: ALA-PDT	Correlation to wound mean volume reduction	Unclear, around 3 w	Biopsy	IHC	TGF-β, TNF-α (TNF-α reported for mastcells)	↑ TGF-β correlated to healing
Harris 1995 [9], UK	Cross-sectional	Healing vs. non-healing	NA	WF	ELISA, bioassay	FGF-2, GM-CSF, IL-1α, IL-1α bio,	↔

	study					IL-1β bio, IL-6 bio, PDGF-AB,	
He 1997 [10], UK	Experimental study (reperfusion injury)	Correlation to healing	3 h 20 min	Serum from leg and arm	Sandwich enzyme immunoassay	IL-1β, IL-1RA, IL-6, TNF-α	↔
Hodde 2020 [11], USA	Clinical trial: SIS wound matrix	Healed vs. non-healed: w 12	Up to 12 w	WF	Luminex ELISA: TGF-β1	GM-CSF, IFN-γ, IL-1β, IL-2, IL-4, IL-5, IL-8, IL-10, IL-12(p70), IL-13, TGF-β1, TNF-α	↓ IL-8 and TNF-α from baseline in healed. IL-1β showed a trend towards reduction in healed but increase in non-healed. ↑ TGF-β1 from baseline in healed vs. non-healed.
Krejner 2017 [12], Poland	Retrospective cohort study	Healing per week. Poor: <5%, Moderate: 5-10%, Good: 11-15%, Fast: >15%	-4w	Serum	ELISA	IL-6, IL-8, TNF	↔
Lagattolla 1995 [13], UK	Cohort study*	a. Healed vs. non-healed<br>b. Correlation to time to healing	6 mo	Biopsy	ELISA	FGF-2, PDGF-AB, TGF-β1	a. ↑ FGF-2 at the ulcer edge in healed vs. non-healed, but not sign. for ulcer bed.<br>b. Increased TGF-β1 at the wound edge correlated to rapid healing (sign. trend).
Ligi 2016 [14], Italy	Cross-sectional and cohort study	Inflammatory vs. granulating VLU	During admission	WF	Luminex	FGF-2, G-CSF, GM-CSF, IFN-γ, IL-1β, IL-1ra, IL-2, IL-4, IL-5, IL-6, IL-7, IL-8, IL-9, IL-10, IL-12 (p70), IL-13, IL-15, IL-17, PDGF-bb, TNF-α, VEGF	↑ GM-CSF, IL-1β, IL-12p70, IL-10, IL-8, VEGF in inflammatory vs. granulating VLU.<br>↑PDGF-bb in granulating
Ligi 2017 [15], Italy	Cross-sectional study and cohort study*	Inflammatory vs. granulating VLU	NA	WF	Luminex	TGF-β1, TGF-β2, TGF-β3	↑ TGF-β3 in inflammatory vs granulating VLU
McQuilling 2021 [16], USA	Clinical trial: Amniotic membrane	a. Healing vs. non-healing (>85% vs. < 85% closure at 12 w)<br>b. Correlation between cytokine level and percentage area change	12 w	WF	Multiplex MAP arrays	EGF, FGF-2, G-CSF, GM-CSF, IFN-γ, IL-1α, IL-1β, IL-1ra, IL-2, IL-3, IL-4, IL-5, IL-6, IL-7, IL-8, IL-9, IL-10, IL-12p40, IL-12p70, IL-13, IL-15, IL-17, PDGF-AA, PDGF-BB, TNF-α, TNF-β, TGF-α, TGF-1β, TGF-β2, TGF-β3, VEGF	a. ↑ IL-1ra, IL-1α, IL-2, IL-3, IL-9, TNF-β in non-healing, evaluated at all time-points.<br>b. ↓ IL-1ra, IL-7 and IL-8, and ↑EGF correlated to size reduction.
Murphy 2002 [17], Ireland	Cohort study	Healing vs. non-healing (before & after compression)	12 w	Serum, from ulcer leg	ELISA	TNF-α, VEGF	↑ VEGF and TNF-**α** in non-healing. Reduction in the levels to below control values with compression, correlating with healing.
Mwaura 2006 [18], Ireland	Cohort study	Healing vs. non-healing:	8 w	WF, biopsy	ELISA, IHC	PDGF-AA	↑ PDGF-AA in WF and biopsy of healing VLU. PDGF-AA

		<20% vs. >20% decrease in ulcer area					activity is associated with ulcer healing.
Pukstad 2010 [19], UK	Cohort study	Healing vs. non-healing	8 w, or healing	WF	Antibody glass array, TNF-α bioassay	40 cytokines analyzed. Results presented on IL-1α, IL-1β, IL-6R, IL-8, sTNFR-I, sTNFR-II,. TNF-α from monocytes	No test of significance. Healing associated with reduction in IL-1α and IL-1β. Increased IL-8 was seen in healing wounds, suggesting that increasing levels of IL-8 in chronic wound fluids is associated with healing. In non-healing wounds IL-8 decreased with time.
Sadler 2012 [20], Australia	Clinical trial: High vs. low dose doxycycline	Association between cytokine level w 4 and percentage reduction in ulcer area	4 w	WF	ELISA	TNF-α	↔
Senet 2003 [21], France	RCT: Platelets vs. placebo	Healing vs. non-healing	16 w	WF	ELISA	IL-8, KGF, VEGF	↑ IL-8 in non-healing at the end of the study, but not at the beginning. Healing is associated with a decrease in IL-8.
Serra 2013 [22], Italy	RCT: Minocycline vs. control	High-healing vs. slow-healing (≥1 vs. <1 cm^2/w)	Median: 13 mo	WF, plasma	ELISA	VEGF	↑ VEGF in slow-healing ulcers, in plasma and WF for patients receiving minocycline. Unclear if this only refers to the experimental arm or also the control.
Serra 2015 [23], Italy	RCT: Doxycycline vs. control	High-healing vs. slow-healing (≥1 vs. <1 cm^2/w)	Median: 13 mo	WF, plasma	ELISA	VEGF	↑ VEGF in slow-healing ulcers in plasma and WF for pt receiving doxycyline. Unclear if this only refers to the experimental arm or also the control.
Stacey 2019 [24], Australia	Cohort study	Healing vs. non-healing *(Area change over 3 w to determine the healing status each week)*	Up to 13 w	WF	Multiplex ELISA	G-CSF, GM-CSF, IFN-γ ("TFN-g"), IL-1α, IL-1β, IL-1Ra, IL-2, IL-4, IL-5, IL-6, IL-7, IL-8, IL-10, IL-11, IL-12p40, IL-12p70, IL-13, IL-15, IL-16, IL-17, M-CSF, PDGF-BB, TNF-α, TNF-β, TNF-RI, TNF-RII	↑ GM-CSF and IL-16, and ↓ IL-6 and PDGF-BB, in non-healing by univariable analysis. Multivariable logistic regression: ↑ GM-CSF only remaining significant, OR 126.5, ROC: 0.92. GM-CSF is a predictive biomarker of nonhealing, and have cutoffs which can be used to differentiate between healing and non-healing VLU. Predictive factor for healing.
Tian 2003 [25], Australia	Cohort study	Healing vs. non-healing	2 w	Biopsy	IHC	EGF, FGF-2, IL-1α, IL-6, PDGF-A, TGF-β1, TNF-α, TNF-RI, VEGF	↑ All cytokines in keratinocytes in the adjacent intact skin in non-healing. ↑ VEGF and PDGF-A in keratinocytes at the ulcer edge in non-healing, but not for other cytokines.
Trengove 2000 [26], Australia	Cohort study	a. Healing vs. non-healing<br>b. Ulcer size change	2 w	WF	ELISA & bioassay	EGF, FGF-2, IL-1α, IL-1β, IL-1 bio, IL-6, IL-6 bio, PDGF, TNF-α, TGF-β1	a.↑ IL-1α, IL-1β, IL-1 bio, IL-6 bio and TNF-α in non-healing.<br>b. ↔ No relationship between change in ulcer size and cytokine levels

Wallace 1998 [27], Australia	Cohort study	Healing vs. non-healing	2 w	WF	ELISA & bioassay	TNF-α, TNF-α bio, sTNF-α-R p75, sTNF-α-R p55	↑ TNF-α and sTNF-α-R p75 in non-healing
Wiegand 2017 [28], USA	RCT: Ultrasound vs. control	Correlation to<br>a. Wound size reduction<br>b. Responder vs. non-responder (>40% vs. ≤40% area reduction w 4)	4 w	WF<br>Biopsy	Luminex<br>TGF-β: IHC	IL-1β, IL-6, IL-8, IL-10, TNF-α, TGF-β (the last, biopsy)	a. ↓ IL-6 correlated to percentage wound reduction (prediction of healing). IL-8 showed a similar trend. In contrast, increases of TNF-α and IL-1β was linked to higher percentage area reduction (significance unclear).<br>b. Data challenging to extract, test of significance unclear. Cytokine levels appears different between the treatment groups, and changes with time during w 2 and w 4, e.g. IL-8 declined in responders, but increased in non-responders over time.

↑ Increased, ↓ decreased, ↔ no difference, *assumed. Abbreviations: ALA-PDT = Aminolevulinic acid photodynamic therapy; cDNA = Complementary DNA; CFU = Colony forming unit(s); ELISA = Enzyme-linked immuno sorbent assay; FAP = Frozen allogenic plasma; IHC = Immunohistochemistry; Luminex = Multiplex Assay; Mo = Months; NA = Not applicable; NLFU = Noncontact low-frequency ultrasound; NR = Not reported; PBMC = Peripheral blood mononuclear cells; Pt = Patients; QRT-PCR = Quantitative reverse transcriptase polymerase chain reaction; RT-PCR = Reverse transcriptase polymerase chain reaction; VAS = Visual analogue scale; VLU = Venous leg ulcer(s), WF = Wound fluid

References

1. Beidler, S.K.; Douillet, C.D.; Berndt, D.F.; Keagy, B.A.; Rich, P.B.; Marston, W.A. Inflammatory cytokine levels in chronic venous insufficiency ulcer tissue before and after compression therapy. *J Vasc Surg* **2009**, *49*, 1013-1020, doi:10.1016/j.jvs.2008.11.049.
2. Charles, C.A.; Tomic-Canic, M.; Vincek, V.; Nassiri, M.; Stojadinovic, O.; Eaglstein, W.H.; Kirsner, R.S. A gene signature of nonhealing venous ulcers: potential diagnostic markers. *J Am Acad Dermatol* **2008**, *59*, 758-771, doi:10.1016/j.jaad.2008.07.018.
3. Drinkwater, S.L.; Burnand, K.G.; Ding, R.; Smith, A. Increased but ineffectual angiogenic drive in nonhealing venous leg ulcers. *J Vasc Surg* **2003**, *38*, 1106-1112, doi:10.1016/s0741-5214(03)01053-x.
4. Escandon, J.; Vivas, A.C.; Perez, R.; Kirsner, R.; Davis, S. A prospective pilot study of ultrasound therapy effectiveness in refractory venous leg ulcers. *Int Wound J* **2012**, *9*, 570-578, doi:10.1111/j.1742-481X.2011.00921.x.
5. Filkor, K.; Németh, T.; Nagy, I.; Kondorosi, É.; Urbán, E.; Kemény, L.; Szolnoky, G. The expression of inflammatory cytokines, TAM tyrosine kinase receptors and their ligands is upregulated in venous leg ulcer patients: a novel insight into chronic wound immunity. *Int Wound J* **2016**, *13*, 554-562, doi:10.1111/iwj.12473.
6. Fivenson, D.P.; Faria, D.T.; Nickoloff, B.J.; Poverini, P.J.; Kunkel, S.; Burdick, M.; Strieter, R.M. Chemokine and inflammatory cytokine changes during chronic wound healing. *Wound Repair Regen* **1997**, *5*, 310-322, doi:10.1046/j.1524-475X.1997.50405.x.
7. Gohel, M.S.; Windhaber, R.A.; Tarlton, J.F.; Whyman, M.R.; Poskitt, K.R. The relationship between cytokine concentrations and wound healing in chronic venous ulceration. *J Vasc Surg* **2008**, *48*, 1272-1277, doi:10.1016/j.jvs.2008.06.042.

8. Grandi, V.; Bacci, S.; Corsi, A.; Sessa, M.; Puliti, E.; Murciano, N.; Scavone, F.; Cappugi, P.; Pimpinelli, N. ALA-PDT exerts beneficial effects on chronic venous ulcers by inducing changes in inflammatory microenvironment, especially through increased TGF-beta release: A pilot clinical and translational study. *Photodiagnosis Photodyn Ther* **2018**, *21*, 252-256, doi:10.1016/j.pdpdt.2017.12.012.
9. Harris, I.R.; Yee, K.C.; Walters, C.E.; Cunliffe, W.J.; Kearney, J.N.; Wood, E.J.; Ingham, E. Cytokine and protease levels in healing and non-healing chronic venous leg ulcers. *Exp Dermatol* **1995**, *4*, 342-349.
10. He, C.F.; Cherry, G.W.; Arnold, F. Postural vasoregulation and mediators of reperfusion injury in venous ulceration. *J Vasc Surg* **1997**, *25*, 647-653, doi:10.1016/s0741-5214(97)70290-8.
11. Hodde, J.P.; Hiles, M.C.; Metzger, D.W. Characterization of the local wound environment following treatment of chronic leg ulcers with SIS wound matrix. *J Tissue Viability* **2020**, *29*, 42-47, doi:10.1016/j.jtv.2019.12.003.
12. Krejner, A.; Litwiniuk, M.; Grzela, T. LL-37 but Not 25-Hydroxy-Vitamin D Serum Level Correlates with Healing of Venous Leg Ulcers. *Arch Immunol Ther Exp (Warsz)* **2017**, *65*, 455-461, doi:10.1007/s00005-016-0423-9.
13. Lagattolla, N.R.; Stacey, M.C.; Burnand, K.G.; Gaffney, P.G. Growth factors, tissue and urokinase-type plasminogen activators in venous ulcers. *Ann Cardiol Angeiol (Paris)* **1995**, *44*, 299-303.
14. Ligi, D.; Mosti, G.; Croce, L.; Raffetto, J.D.; Mannello, F. Chronic venous disease - Part I: Inflammatory biomarkers in wound healing. *Biochim Biophys Acta* **2016**, *1862*, 1964-1974, doi:10.1016/j.bbadis.2016.07.018.
15. Ligi, D.; Croce, L.; Mosti, G.; Raffetto, J.D.; Mannello, F. Chronic Venous Insufficiency: Transforming Growth Factor-β Isoforms and Soluble Endoglin Concentration in Different States of Wound Healing. *Int J Mol Sci* **2017**, *18*, doi:10.3390/ijms18102206.
16. McQuilling, J.P.; Carter, M.J.; Fulton, J.A.; Patel, K.; Doner, B.; Serena, T.E.; Mowry, K.C. A prospective clinical trial evaluating changes in the wound microenvironment in patients with chronic venous leg ulcers treated with a hypothermically stored amniotic membrane. *Int Wound J* **2021**, *19*, doi:10.1111/iwj.13606.
17. Murphy, M.A.; Joyce, W.P.; Condron, C.; Bouchier-Hayes, D. A reduction in serum cytokine levels parallels healing of venous ulcers in patients undergoing compression therapy. *Eur J Vasc Endovasc Surg* **2002**, *23*, 349-352, doi:10.1053/ejvs.2002.1597.
18. Mwaura, B.; Mahendran, B.; Hynes, N.; Defreitas, D.; Avalos, G.; Adegbola, T.; Adham, M.; Connolly, C.E.; Sultan, S. The impact of differential expression of extracellular matrix metalloproteinase inducer, matrix metalloproteinase-2, tissue inhibitor of matrix metalloproteinase-2 and PDGF-AA on the chronicity of venous leg ulcers. *Eur J Vasc Endovasc Surg* **2006**, *31*, 306-310, doi:10.1016/j.ejvs.2005.08.007.
19. Pukstad, B.S.; Ryan, L.; Flo, T.H.; Stenvik, J.; Moseley, R.; Harding, K.; Thomas, D.W.; Espevik, T. Non-healing is associated with persistent stimulation of the innate immune response in chronic venous leg ulcers. *J Dermatol Sci* **2010**, *59*, 115-122, doi:10.1016/j.jdermsci.2010.05.003.
20. Sadler, G.M.; Wallace, H.J.; Stacey, M.C. Oral doxycycline for the treatment of chronic leg ulceration. *Archives of dermatological research* **2012**, *304*, 487-493, doi:10.1007/s00403-011-1201-5.
21. Senet, P.; Bon, F.X.; Benbunan, M.; Bussel, A.; Traineau, R.; Calvo, F.; Dubertret, L.; Dosquet, C. Randomized trial and local biological effect of autologous platelets used as adjuvant therapy for chronic venous leg ulcers. *J Vasc Surg* **2003**, *38*, 1342-1348, doi:10.1016/s0741-5214(03)00908-x.
22. Serra, R.G., R.; Buffone, G.; Gallelli, L.; De Franciscis, S. The effects of minocycline on extracellular matrix in patients with chronic venous leg ulcers. *Acta Phlebologica* **2013**, *14*, 99-107.
23. Serra, R.; Gallelli, L.; Buffone, G.; Molinari, V.; Stillitano, D.M.; Palmieri, C.; de Franciscis, S. Doxycycline speeds up healing of chronic venous ulcers. *Int Wound J* **2015**, *12*, 179-184, doi:10.1111/iwj.12077.
24. Stacey, M.C.; Phillips, S.A.; Farrokhyar, F.; Swaine, J.M. Evaluation of wound fluid biomarkers to determine healing in adults with venous leg ulcers: A prospective study. *Wound Repair Regen* **2019**, *27*, 509-518, doi:10.1111/wrr.12723.

25. Tian, Y.W.; Stacey, M.C. Cytokines and growth factors in keratinocytes and sweat glands in chronic venous leg ulcers. An immunohistochemical study. *Wound Repair Regen* **2003**, *11*, 316-325, doi:10.1046/j.1524-475x.2003.11502.x.
26. Trengove, N.J.; Bielefeldt-Ohmann, H.; Stacey, M.C. Mitogenic activity and cytokine levels in non-healing and healing chronic leg ulcers. *Wound Repair Regen* **2000**, *8*, 13-25.
27. Wallace, H.J.; Stacey, M.C. Levels of tumor necrosis factor-alpha (TNF-alpha) and soluble TNF receptors in chronic venous leg ulcers--correlations to healing status. *J Invest Dermatol* **1998**, *110*, 292-296, doi:10.1046/j.1523-1747.1998.00113.x.
28. Wiegand, C.; Bittenger, K.; Galiano, R.D.; Driver, V.R.; Gibbons, G.W. Does noncontact low-frequency ultrasound therapy contribute to wound healing at the molecular level? *Wound Repair Regen* **2017**, *25*, 871-882, doi:10.1111/wrr.12595.
